# Low-Cost Radon Detector with Low-Voltage Air-Ionization Chamber

**DOI:** 10.3390/s19173721

**Published:** 2019-08-28

**Authors:** Filip Studnička, Jan Štěpán, Jan Šlégr

**Affiliations:** 1 Department of Physics, Faculty of Science, University of Hradec Králové, Rokitanského 62, 500 03 Hradec Králové, Czech Republic; 2 Center of Advanced Technology, Faculty of Science, University of Hradec Králové, Rokitanského 62, 500 03 Hradec Králové, Czech Republic

**Keywords:** charge-carrier processes, ionizing radiation sensors, wireless sensor networks

## Abstract

This paper describes the design of a low-cost radon detector that can easily be fabricated in large quantities for the purposes of earthquake prediction. The described detector can also be used for monitoring radon levels in houses because high radon levels pose a great health risk. A very simple air-ionization chamber for alpha particles was used, considering the experimental results. Chamber current-sensing circuitry is also suggested, and an Internet of Things (IoT) sensor grid is described. The main advantages of this detector are the low cost, low power consumption, and complete elimination of high-voltage power sources. The minimum detectable activity achieved with the proposed detector for one measurement was around $50\;\mathrm{Bq}\cdot m^{-3}$, with time of measurement comparable to that featured on commercial devices, while the price of the described detector is one order of magnitude lower.

## 1. Introduction

Earthquake prediction (a branch of seismology concerned with the specification of the time, location, and magnitude of future earthquakes [1]) uses several physical parameters to assess the probability of an impending earthquake. It was shown that the earthquake-preparation process relates to a complex chain of physical and chemical transformations within Earth’s crust in the environs of the epicenter [2]. These processes can be accompanied by measurable changes in several physical parameters, such as electric resistivity, geoelectric potentials, or concentration of radon gas. There is a known association between earthquake occurrence and radon emissions from the soil in the vicinity of the epicenter called the earthquake-preparation zone [3]. For the diameter of the earthquake-preparation zone, the following empirical expression is used [4]:

$$R=10^{0.43M}\;\mathrm{km},$$

where M is the magnitude of the earthquake. This method of earthquake prediction is currently investigated (see, e.g., [5] or review study [6]), but detection requires relatively expensive equipment that prevents the continuous monitoring of large areas. The paper aims to describe an inexpensive radon detector that can be used to cover a larger area and communicate through the Internet of Things (IoT). Similar detectors have been proposed in the past, but they used high voltage to collect ionized radon progeny [7] or added measurement and IoT communication capabilities to commercially available low-cost detectors [8].

For the aim of verifying such earthquake-prediction studies on solid statistical ground, one must create a wide network of cheap, compact, and high-sensitivity radon detectors. Several hundred devices must be deployed at key points where the earthquake may potentially appear. These detectors should have low power consumption because of long-term measurements, and withstand conditions in wells and, in some cases, in mines. The sensitivity threshold should be around $50\;\mathrm{Bq}\cdot m^{-3}$, but precise calibration is usually not needed; in the case of earthquake prediction, only relative changes in radon concentration are essential.

Very important is the time needed to complete one measurement, as radon concentration can change in an order of hours, so reaction time of the detector in tens of minutes is necessary.

## 2. Materials and Methods

Of all the natural and artificial sources of ionizing radiation, radon gas is a significant contributor. Radon monitoring is thus essential not only for earthquake prediction, but also to identify health risks to the public. There are several off-the-shelf detectors that work on various principles that effectively and affordably monitor radon-gas levels. The most widely used detectors for general indoor radon-level measurements are based on the accumulative technique [9]. Activated-charcoal types are used when the average exposition must be determined, and if we can assume stable radon concentration.

There are also alpha-track detectors or solid-state nuclear track detectors [10] based on a detecting material that is impacted by alpha particles that produce so-called latent alpha tracks—microscopic areas of damage that are counted (either manually or automatically) after the exposure period. Passive detectors also include electret systems for integral dosimetry [11]. Passive detectors and electret systems are only suitable for long-term measurement (on a time scale of days to weeks).

For real-time or nearly real-time radon monitoring, different methods must be used, usually using continuous radon monitors [12].

Continuous radon detectors collect radon progeny from the collection volume (into which the radon diffuses through air filter). In most cases, polonium-218 is collected, and subsequent radioactive decay is detected. Radon-222 decays to polonium-218 through alpha decay with a half-life of 3.82 days, which further decays to lead-214 with a half-life of 3.10 min:

$${}_{\;86}^{222}\mathrm{Rn}\to_{\;84}^{218}\mathrm{Po}{+}_{\;2}^{\;4}\mathrm{He},$$

$${}_{\;84}^{218}\mathrm{Po}\to_{\;82}^{214}\mathrm{Pb}{+}_{\;2}^{\;4}\mathrm{He},$$

Because radon’s progeny can be positively charged [13], negative high voltage is applied to the semiconductor detector. It is necessary to apply high voltage to reduce the influence of moisture (positively charged radon-decay products can be neutralized to water vapor [14]). The device, therefore, uses stabilized voltage of a few thousand volts. Active systems often use fans to increase the volume of sampled air to improve counting statistics. On the other hand, free-air detectors (either using ionization chambers or electrostatic collection detectors) do not use fans.

Earthquake-forecasting studies require massive use of fast detectors (able to perform one measuring cycle under ten minutes) and can cooperate online. Hence, the construction of a continuous detector with an ionization chamber is described. The low cost of the described design allows detectors to be mass-produced to cover large areas where radon precursors are studied.

### 2.1. Ionization Chambers

The operation principle of an ionization chamber is straightforward. If a charged particle is passing through the volume of a gas or liquid, through ionization, electron–ion pairs are created. Ions can be directly created by the incident particle, or by the δ-electrons when the energy of the primary particle is transferred to the electron that acquires enough energy to produce further ionization [15]. If an electric field is applied to the electrodes, electrons, and ions drift to them. A typical example is the Geiger–Müller tube [16].

Ionization chambers are also used in commercial smoke detectors with americium-241, where the ionization current from an alpha source decreases due to the presence of smoke in the sampled air.

Minimum energy *W* must be transferred from the incident particle to create at least one electron–ion pair. For alpha particles in the air, the *W*-value is 35.1 eV/pair [17].

For the design of the ionization chamber, another quantity is important: specific ionization is defined as the number of ion pairs that a particle produces per unit distance. This quantity expresses the density of ionization along a track:

$$SI=\frac{dE}{Wdx},$$

where term d*E*/d*x* is linear energy transfer. For ~5 MeV energy particles originating from the decay of polonium-218, we can calculate the specific ionization $(1.23\times 10^{6}\;\mathrm{eV}\cdot {\mathrm{cm}}^{-1})/\left(35\;\mathrm{eV}\right)=35,000\;{\mathrm{cm}}^{-1}$, since the stopping power of air is 1.23 MeV/cm for 5 MeV alpha particles [18], so these particles stop within a few centimeters.

Other geometric properties are governed by the required electric field values. In most cases, cylindrical geometry with an anode wire is used with electrical-field intensity

$$E\left(r\right)=\frac{U_{0}}{r\ln\frac{r_{2}}{r_{1}}},$$

where *U*_0_, voltage between electrodes; *r*_1_, anode-wire diameter; and *r*_2_ diameter of cylindrical cathode.

From an electrical point of view, the ion chamber behaves like a parallel connection of resistor and capacitor, so the response to initial charge-giving voltage *U*_0_ is

$$U\left(t\right)=U_{0}e^{-\frac{t}{RC}}.$$

If the RC constant is long enough to collect all ion pairs, the maximum amplitude of the voltage pulse can be derived as

$$U_{\max}=\frac{n_{0}e}{C},$$

where *n*_0_ is the number of ion pairs, *e* is the elementary charge, and *C* is the capacitance of the cylindrical ion chamber. This result is independent of the initial charge position.

Capacitance *C* of the system described here was around 100 pF, so for the voltage, change is $4.6\times 10^{-5}$ V. That is why a sensitive, low-noise preamplifier is needed.

Ionization charge can be calculated as follows:

$$\Delta Q=\frac{n_{0}}{U_{0}}\int_{}^{}E(r)dr=\frac{n_{0}}{\ln\frac{r_{2}}{r_{1}}}\ln\frac{r_{1}}{l_{0}},$$

where *l*_0_ is typical ionization length.

**Figure 1:**
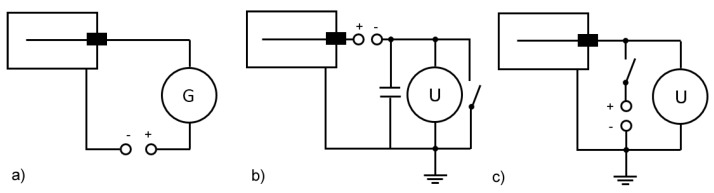
Methods of ionization-current measurements: (**a**) Direct, (**b**) charging, and (**c**) discharging method.

If the intensity of radiation is sufficiently high, we can directly measure the current. If the chamber works in the Townsend discharge region of the current–voltage curve, current is proportional to intensity (Figure 1a). The charging method (Figure 1b) uses the ionization current to charge the capacitor in the outer circuit. Voltage across capacitor plates is then measured, which is proportional to the dose of ionizing radiation that passed through the chamber, making this circuit a dosimeter. The discharging method (Figure 1c) is based on the opposite principle: the ionization chamber is temporarily connected to the battery, so the system acquires a charge given by the capacitance of the system

Q=CU.

After voltage disconnection, the chamber starts to partially discharge due to ionizing radiation. If, during time *t*, voltage drops by *U*, from known capacity *C* we can calculate charge *Q* carried away by the emitted ions

ΔQ=CΔU.

Generally available air-chamber dosimeters (using a cylindrical capacitor filled with air initially charged to high potential *U*_0_) work in this fashion.

It should be noted that because of a very small ionization current (less than 10${}^{-12}$ A), the insulation of the central electrode must be at least 10^16^ Ω to prevent charge leakage.

### 2.2. Radon-Progeny Alpha-Detector Construction

The proposed low-cost detector uses one wire-ionization chamber described above (in contrast to older designs such as [19] that use several wire electrodes) and eliminates the need for a high-voltage source. The potential needed to help the collection of radon progeny was 9–18 volts in our case, hence “low-voltage” air-ionization chamber.

**Figure 2:**
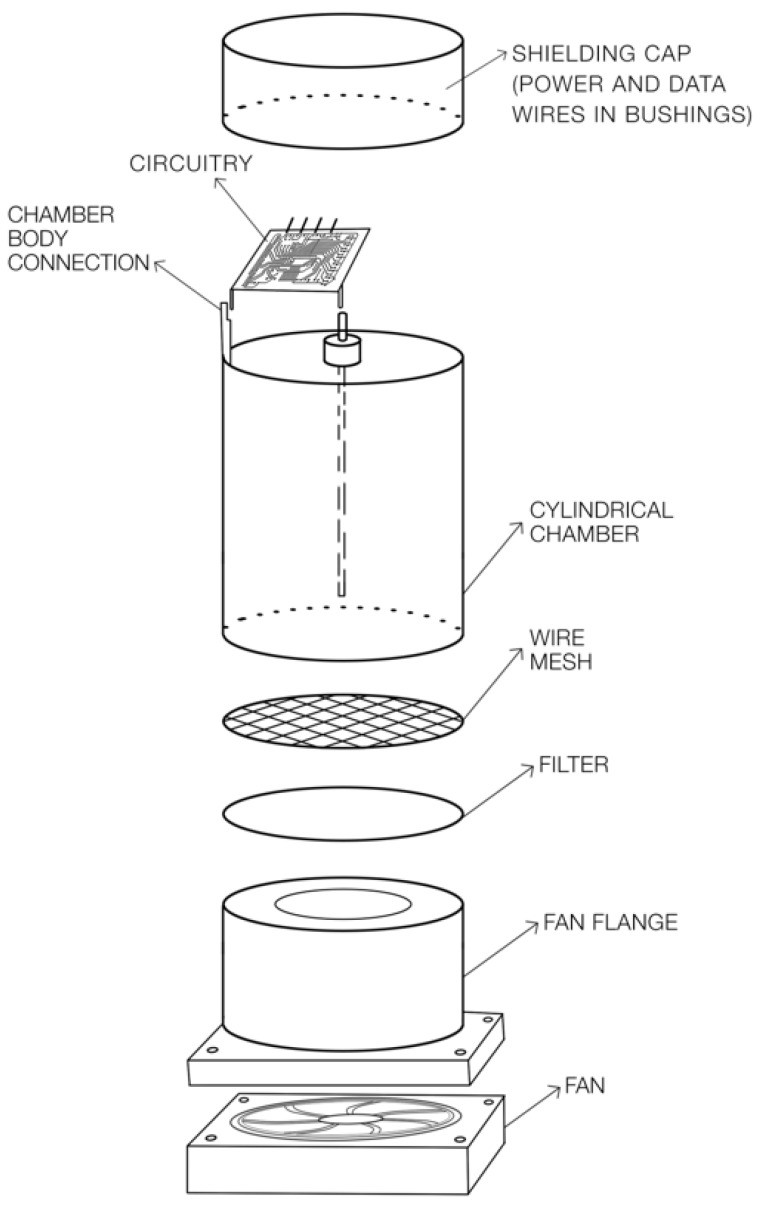
Construction of open air-ionization-chamber radon detector.

The detector (see Figure 2) consisted of a cylindrical metal chamber of 4${}^{\prime \prime }$ (10 cm) diameter and 5.5${}^{\prime \prime }$ (14 cm) height. A 4${}^{\prime \prime }$ (10 cm) wire was suspended inside the chamber using Teflon rod as an insulator. Sensing circuitry was connected between the rod and chamber body under the shielding cap to prevent static electricity from altering measurements. The lower side was closed with wire mesh to complete the shielding, and a porous air filter with a weight of 100–200 $g\cdot m^{-2}$ (impregnated to improve the resistance to moisture) was placed over the fan flange. Using the fan, the airflow in the thin slit between wire mesh and fan flange could flow through the filter, depositing desired particles in the filter.

For data acquisition and online data transfer, the Arrow SmartEverything Fox 3 (MC27561-FOX, Arrow Electronics, Centennial, CO, USA) experiment board [20] was used. It is a flexible prototyping platform for IoT applications. SmartEverything combines SigFox, Bluetooth, and Near-Field Communication (NFC) wireless technologies with GPS and a suite of embedded sensors. In this case, temperature, air pressure, and air-humidity sensors were used to calibrate the measured data and the SigFox IoT protocol to send the results to the Cloud.

Two methods of ionization-current measurement were adopted: Direct (Figure 1a) and discharge (Figure 1c).

#### 2.2.1. Direct Measurement Method

In the first case, the input resistance of the analog-digital-converter (ADC) of the Arrow SmartEverything board was used to measure the current, which was amplified in the simplest case by a Darlington transistor or with a specialized electrometer operational amplifier (that, however, could not be regarded as low-cost). In either case, precise absolute measurement of picoampere currents is not needed—we needed to monitor the time changes of such currents and compare the actual values to previous ones. The described design of the chamber was very sensitive to static electricity, and affected by vibration and nearby movement. Circuitry had to be shielded, and movement in the detector vicinity had to be kept to a minimum, which is not a significant problem in earthquake detection (detectors are usually placed in a stable environment).

**Figure 3:**
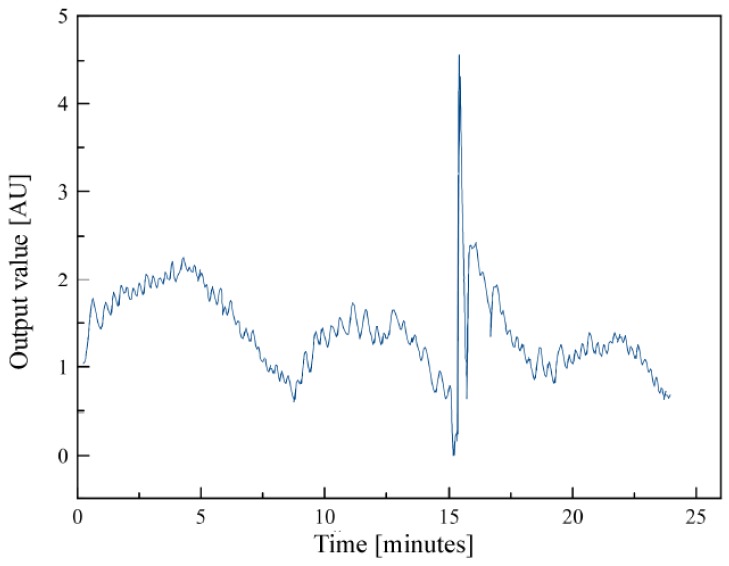
Sensing-circuit output voltage (not calibrated) vs. time for direct method.

Radon measurement was performed using a fan and porous material (i.e., filter paper) placed in front of the ionization chamber (see Figure 2). When the fan was turned on, air started to flow through the filter and radon progenies started to be deposited. As radon and its progeny decayed, the ionization current increased. At some point, there was enough radioactive material on the filter that the rate of decay equaled the rate of collection. This dynamical equilibrium point is seen as a maximum in the curve in Figure 3. If the fan was stopped, exponential decay of the radioactive material on the filter occurred, and the ionization current returned to the baseline. It is advantageous to use averaging of the measured voltage values for this method.

#### 2.2.2. Discharge Measurement Method

**Figure 4:**
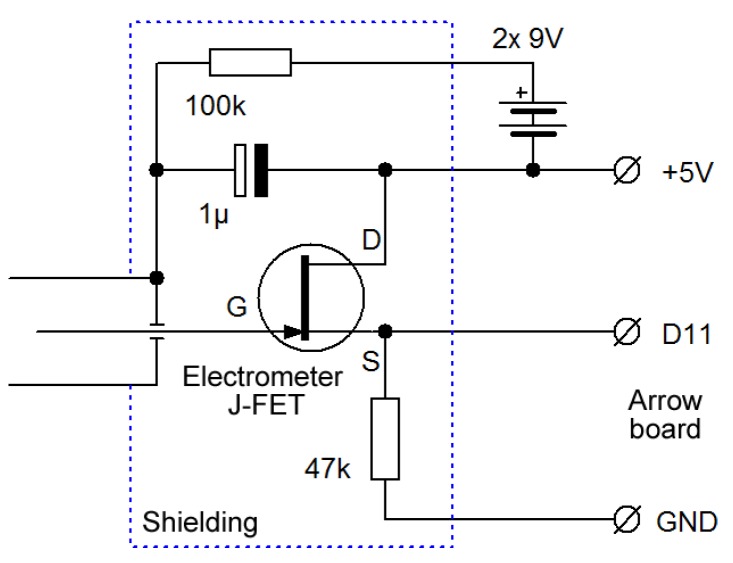
Simplest concept schematics of sensing circuitry—discharge method.

When implementing the discharge method, we could take advantage of the fact that the microcontroller pin could operate as output (in the High or Low logical state) or as digital input, which has high impedance. Digital inputs of the previously mentioned Arrow SmartEverything board were used. The key was to measure the voltage-drift rate on the ionization chamber’s sense wire. An electrometer-grade JFET transistor could be used to buffer voltage on a sense wire inside the chamber, and a microprocessor measured the rate of change of that voltage, periodically discharging the wire through the JFET’s gate-source junction (see Figure 4). The chamber was discharged at the beginning by momentarily connecting the JFET source to ground. Then, the microcontroller pin was switched to input, and its state (which was initially Low with discharged capacity) was read. Then, the time was measured until voltage on the input pin reached the value needed to switch the logic level to High. The more the current flowed in the chamber (the higher the intensity of the ionizing radiation was), the shorter the time was. Positive ions in the chamber produced by radiation were attracted to the central negatively charged wire. When voltage reached the logic threshold, the chamber wire discharged through the gate diode to within a few hundred millivolts of ground. The microprocessor then waited a fixed amount of time (200 ms), needed to discharge the central electrode, and made another measurement. As we can see, this case is the discharging method depicted in Figure 1c. The SigFox board program measured the time needed to reach the High level, which was the time needed to achieve dynamic equilibrium. With increasing radon concentrations, this period shortens because there is more ionization of the air inside the chamber.

From these data, radon-flux changes can be calculated, calibrating the period against a commercially available radon detector.

### 2.3. Data Acquisition and IoT Connectivity

**Figure 5:**
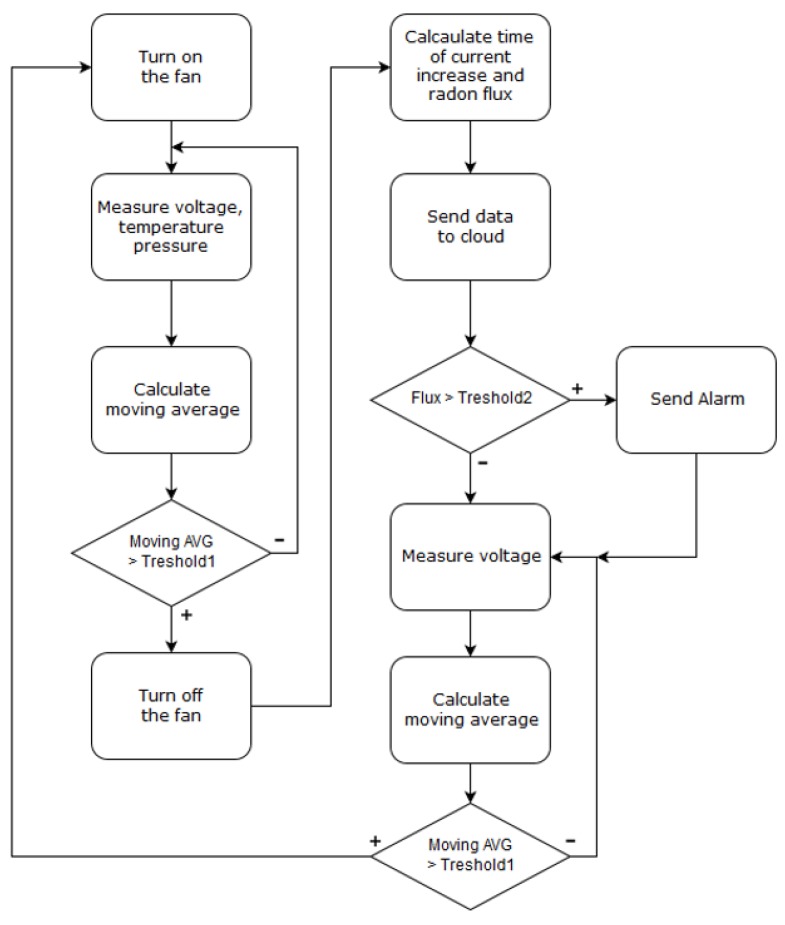
Firmware flowchart.

Using the direct measurement method, after the fan was turned on, voltage was repeatedly measured, and the moving average of the last 50 values began to be calculated (see Figure 5). When the moving average reached the threshold for several consecutive measurements, the fan was stopped and the time of current increase was calculated, from which radon flux was derived.

There is an empirical formula for first-order ionization-current correction for ambient temperature and atmospheric pressure [21]

$$I_{\mathrm{norm}}=I_{t,p}\cdot \left(1+0.00367t\right)\frac{101,325}{p},$$

where *I*_norm_ is the ionization current under normal conditions, *t* is the ambient temperature, and *p* the atmospheric pressure. For early-warning earthquake radon grids, detectors are placed in deep mines and drill holes where changes in temperature and pressure are not too large. Data are sent to the Cloud via the SigFox network for further analysis, including battery-voltage level to notify when to replace the battery. If the flux is higher than the set threshold (Threshold2 in Figure 5), an immediate alarm is sent via SigFox network.

Due to the SAMD21 ultra-low-power microcontroller, measurements with the built-in 12-bit ADC and related calculations are not very power-intensive. If the result of each measurement is sent to the Cloud, energy consumption increases for a short period (up to four seconds) to 60 mA. The component that had the highest electricity demand was the air-circulation fan. Even low-power variants consume approximately 120 mA, and if we assume that the fan was in operation for about half of the measuring-cycle time, we could round energy consumption to approximately 15 mAh per cycle. Using a standard AGM battery rated for 18 Ah, we reached a few hundred cycles before the battery was depleted, making battery life about one week. These calculations have been verified in practice. Longer usability can be achieved by using larger-capacity batteries.

## 3. Results

Preliminary results done in the laboratory by means of the direct method showed the need to use digital filtering, and that the device was prone to static electricity (the peak in Figure 3 was caused by human presence around the detector). The influence of these fluctuations in the measured data could be limited by a simple high-pass filter that effectively eliminates high frequencies and leaves only the desired slow changes caused by changes in air conductivity in the detector.

**Figure 6:**
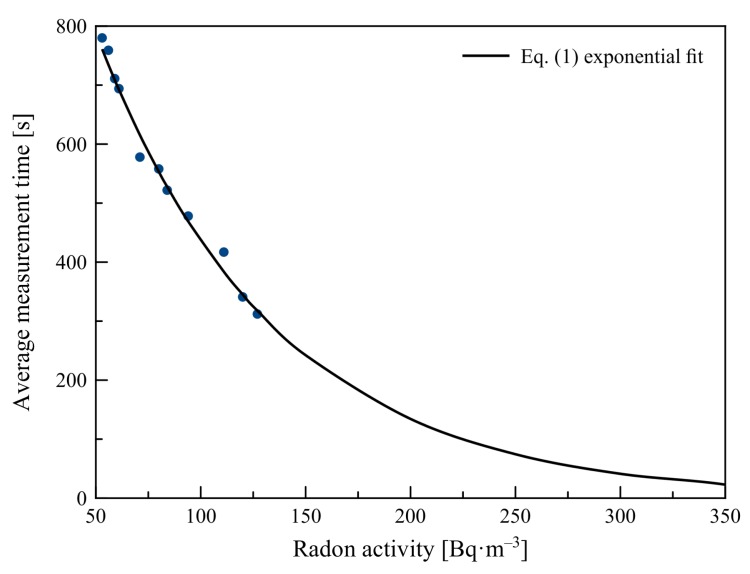
Calibration curve for proposed ionization-chamber radon detector.

It was found during the discharge-method calibration that the sensitivity of the constructed detector was approximately $50\;\mathrm{Bq}\cdot m^{-3}$. Calibration was originally to be done using a polonium alpha emitter to increase air ionization, and measuring it by the constructed detector and a commercially available Safety Siren Pro Series 3 Radon Gas Detector. Finally, measurement was arranged in a way that when the detector showed an increased value in the measured area, the two detectors were enclosed in a vessel where radon-progeny activity gradually decreased due to decay to stable elements. The needed time to charge the ionization chamber was therefore increasing. Thus, it was possible to create a calibration curve that allowed approximate measurements of radon activity (see Figure 6).

Considering the principle of operation of the device, we could assume that Figure 6 shows the declining exponential dependence described by the general equation

y=exp(Ax+B),

that could be linearized in the form

lny=Ax+B.

If we fit a linear function, we obtain form lny=−0.0118x+7.26 with coefficient of determination $R^{2}=0.987$. Thus, the original data could be described by the equation

(1) y=exp(−0.0118x+7.26).

Since the accuracy of the detector used for calibration was ±10$\;\mathrm{Bq}\cdot m^{-3}$, if the average value of measured time reached 10 s, we had to assume that the maximal measurable radon-concentration value was 420 $\mathrm{Bq}\cdot m^{-3}$. This is suitable for measurements of radon levels in residential houses, as well as for measurements of radon emissions from mud volcanoes [22], soil, and wells [23] needed for earthquake forecasting. For applications in mines, where radon concentration could easily reach values above 1.000$\;\mathrm{Bq}\cdot m^{-3}$, another type of detector must be used.

## 4. Conclusions

**Figure 7:**
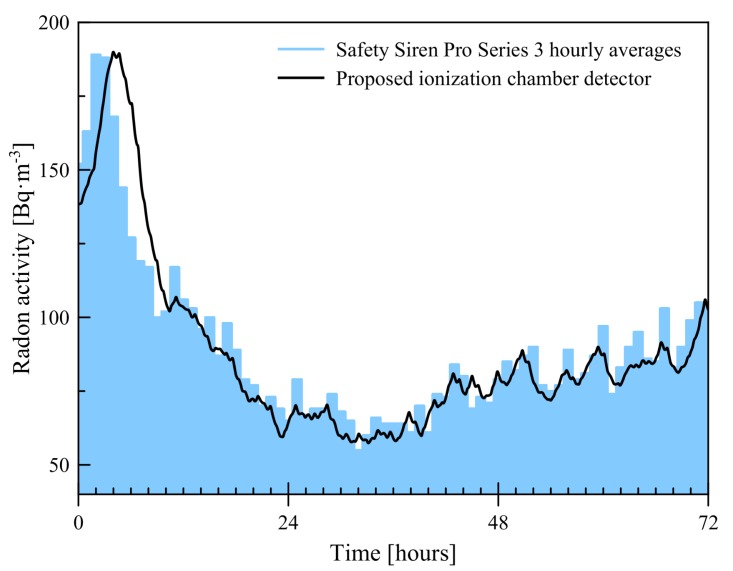
Comparison of proposed detector with commercial device.

We developed a design proposal for an air-filled gaseous detector of alpha particles collected from radon decay that can operate at high gains in the air. It could reliably detect variations in radon concentration, so this information can be used for analysis of the possible correlation between changes in radon levels and forthcoming earthquakes. Figure 7 shows radon concentration measured by the proposed detector with ionization chamber compared with the one measured by commercial device Safety Siren Pro Series 3.

These devices are simple, low-cost, battery-operated, and could be produced in large quantities. Because there were problems with air-humidity corrections (higher humidity inside the chamber lead to charge leaks), silica-gel moisture removers were placed inside the chamber. If soil pores are rich in fluids, the lower level of detection and the chamber’s response could be altered; especially in mines, relative air humidity may be well above 90%. It may be necessary to derive additional correction factors in the same fashion, as described in [24].

Due to the design, the described sensor cannot distinguish between different isotopes. Thoron (${}^{220}$Rn) might have a significant contribution to soil gas depending on the relative concentration of the parent nuclei. This is not a considerable problem, partly because the half-life is 55.6 s and mean life 80 s, and partly because thoron was recently also identified as prospective earthquake precursor [25,26].
